# Implementation of an extended ZINB model in the study of low levels of natural gastrointestinal nematode infections in adult sheep

**DOI:** 10.1186/s12917-016-0723-7

**Published:** 2016-06-10

**Authors:** M. Atlija, J. M. Prada, B. Gutiérrez-Gil, F. A. Rojo-Vázquez, M. J. Stear, J. J. Arranz, M. Martínez-Valladares

**Affiliations:** Departamento de Producción Animal, Universidad de León, Campus de Vegazana s/n, 24071 León, Spain; Institute of Biodiversity, Animal Health and Comparative Medicine, University of Glasgow, Bearsden Road, Glasgow, G61 1QH UK; Department of Ecology and Evolutionary Biology, Princeton University, Princeton, NJ 08540 USA; Instituto de Ganadería de Montaña, CSIC-ULE, 24346 Grulleros, León Spain; Departamento de Sanidad Animal, Universidad de León, Campus de Vegazana s/n, 24071 León, Spain

**Keywords:** Gastrointestinal nematodes, Sheep, Prevalence, Egg count, IgA, ZINB

## Abstract

**Background:**

In this study, two traits related with resistance to gastrointestinal nematodes (GIN) were measured in 529 adult sheep: faecal egg count (FEC) and activity of immunoglobulin A in plasma (IgA). In dry years, FEC can be very low in semi-extensive systems, such as the one studied here, which makes identifying animals that are resistant or susceptible to infection a difficult task. A zero inflated negative binomial model (ZINB) model was used to calculate the extent of zero inflation for FEC; the model was extended to include information from the IgA responses.

**Results:**

In this dataset, 64 % of animals had zero FEC while the ZINB model suggested that 38 % of sheep had not been recently infected with GIN. Therefore 26 % of sheep were predicted to be infected animals with egg counts that were zero or below the detection limit and likely to be relatively resistant to nematode infection. IgA activities of all animals were then used to decide which of the sheep with zero egg counts had been exposed and which sheep had not been recently exposed. Animals with zero FEC and high IgA activity were considered resistant while animals with zero FEC and low IgA activity were considered as not recently infected. For the animals considered as exposed to the infection, the correlations among the studied traits were estimated, and the influence of these traits on the discrimination between unexposed and infected animals was assessed.

**Conclusions:**

The model presented here improved the detection of infected animals with zero FEC. The correlations calculated here will be useful in the development of a reliable index of GIN resistance that could be of assistance for the study of host resistance in studies based on natural infection, especially in adult sheep, and also the design of breeding programs aimed at increasing resistance to parasites.

**Electronic supplementary material:**

The online version of this article (doi:10.1186/s12917-016-0723-7) contains supplementary material, which is available to authorized users.

## Background

Infection by gastrointestinal nematodes (GIN) is common in ruminants worldwide, causing major economic losses due to decreased growth and milk production [1, 2]. Grazing ruminants are infected by a variety of species of GIN with different pathogenicities and geographical distributions [3].

The control of GIN in ruminants is largely based on the use of anthelmintics, combined with grazing management strategies. However, anthelmintic resistance has appeared worldwide [4–6]. In northwest (NW) Spain, a recent survey showed that GIN in 63.6 % of the sampled flocks were resistant to at least one of the most commonly used drugs [7]. The increasing prevalence of anthelmintic resistance has led to the search for alternative control methods, such as selective breeding for resistance to GIN. However, for this purpose, the identification of an appropriate method to measure resistance to infection is necessary, especially in conditions where the worm burden is low. Hence, a sensitive method for detecting infections is needed.

Faecal egg counts (FEC) have been the traditional indicator trait used to assess the level of infection, based on the number of eggs per gram (epg) of faeces, and it is related to both the worm burden and the fecundity of female adults in the host [8–10]. Faecal egg counts have been used to measure genetic resistance to GIN, although in natural infections they can be quite variable both within and between populations [11]. However, FEC are not particularly sensitive and should be interpreted in conjunction with information about the nutritional status, age and management of sheep flocks [12]. As adult sheep are in general more resistant than naïve young animals, their FECs tend to be lower, adding an additional limitation to the sensitivity problem of the technique.

Other phenotypes related to GIN infections, such as the levels of IgA in serum may be taken into account with the goal of defining resistant animals under natural conditions. IgA is a secreted antibody that plays a major role in gut infections. Animals that display high IgA activity have been shown to present lower FEC and shorter adult female *Teladorsagia circumcincta* among experimentally and naturally infected sheep [9, 13, 14].

The distribution of FEC in naturally infected populations is characteristically over-dispersed within domestic and wild animals [15, 16], as well as in human populations [17]. The negative binomial (NB) distribution has been widely used to describe parasite eggs distribution. However, when there are more zero FEC values than expected, zero-inflated negative binomial (ZINB) models are more appropriate [15, 18]. A zero-inflated distribution is a mixture of two distributions and can arise if some animals with zero egg counts have been exposed and are resistant to the infection while other animals with zero egg counts have not been exposed or recently infected e.g. no established worms since the last anthelmintic treatment. Resistant animals tend to have few parasite eggs in their faeces. Due to the McMaster measurement technique, small egg numbers are difficult to detect and will be counted as zero, whether the animal has really zero eggs or just a small number of them. We hypothesize that by exploiting additional information, such as that provided by parasite-specific IgA activity, we could improve the ability to discriminate animals with low level of infection with zero egg counts from unexposed/recently uninfected animals. Therefore, the objective of the study was to determine the prevalence of GIN infections in naturally infected adult sheep showing low levels of infection by combining information from the two widely used indicator traits previously mentioned (FEC and IgA). For this purpose, we applied a ZINB model and extended it to include data from IgA responses. For the subset of animals that were considered as exposed to the infection based on the ZINB model, we calculated the correlations among the two indicator traits related to the infection by GIN (FEC, IgA) and the hidden variable of animal status (i.e. the parameter that determines if the animal has been recently infected or not). The aim was to test whether we could improve the value of mixture and enhance the utility of the ZINB model in animals naturally infected with low doses of parasites.

## Methods

### Study area and animal sampling

**Fig. 1:**
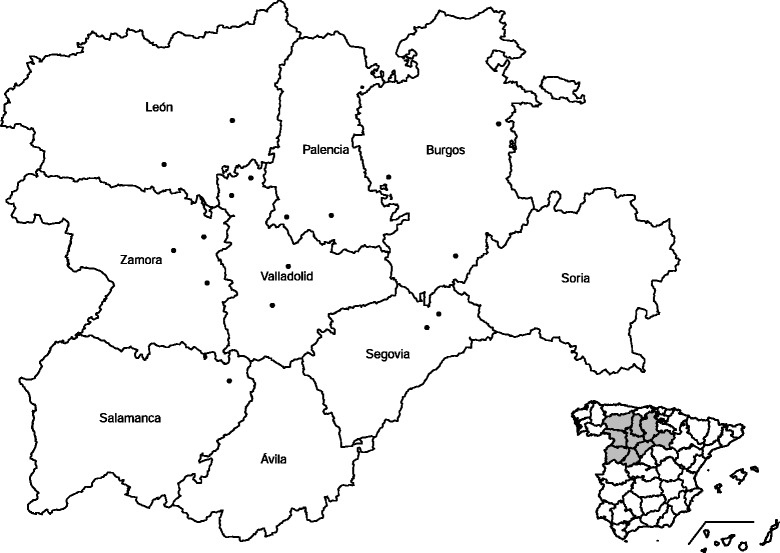
Map of the region of Castilla y Leon (Spain). The map shows the location of the farms where the flocks were sampled. Map created in R using data from www.gadm.org

The study was carried out in the region of Castilla y León, in the NW of Spain, and included 17 commercial dairy flocks distributed in seven out of the nine provinces of the region (Burgos, León, Palencia, Segovia, Valladolid, Salamanca and Zamora) (Fig. 1). In the study area, the flocks are reared under a semi-extensive system in which sheep graze on natural pasture for six hours per day and are kept indoors for the rest of the day. The average size of the sampled flocks was 912, ranging from 302 to 2121 animals per flock.

The survey was conducted from December 2011 to June 2012. This period was extremely dry (Additional file 1). Two conditions had to be met to include a flock in the study: first, the last anthelmintic treatment must have been administered at least 2 months before collecting the samples, and second, the sheep had to be grazing at the time of sampling. The animals included in this study were ewes obtained by artificial insemination from farms belonging to the Selection Nucleus of the National Association of Churra Breeders (ANCHE). Moreover, these animals were a subset of those previously genotyped with the *Illumina* OvineSNP50 BeadChip by [19] which were still alive during the sampling period and for which both phenotypes related to parasite resistance were available. Faecal samples were collected for each ewe directly from the rectum and blood samples were obtained by venipuncture of the jugular vein. Serum samples were stored at -20 °C until processing. This study is based on 529 adult Churra sheep with faecal and blood serum samples. The mean number of sheep sampled per flock was 31 (range: 11–60 individuals). The age of the sheep included in the study varied between 4 and 11 years. All of the sheep were undergoing milking at the time of sampling and were experiencing at least their third lactation.

### Parasitological measures

A modified McMaster technique [20] using zinc sulphate as a flotation solution was used to determine the number of eggs in faeces. The minimum detection limit of this technique was 15 eggs per gram (epg). Faecal egg counts were determined by multiplying the number of eggs observed microscopically (Neggs) by 15.

In each flock, pooled faeces were cultured to recover and identify third-stage larvae (L3) following standard parasitological techniques [20]. A total of 100 L3 were identified per flock to estimate the percentage of each species.

### Titre of IgA

An indirect ELISA was carried out to determine the activity of IgA in the serum, results were scored as optical density (OD). The preparation of somatic antigen from fourth-stage larvae (L4) of *T. circumcincta* was conducted as previously described by [21]. Microtitre plates (Sigma) were coated with 100 μl of PBS containing 2.5 μg/ml of *T. circumcincta* L4 somatic antigen, after which the plates were stored overnight at 4 °C. After discarding their contents, the plates were blocked with 250 μl of PT-Milk (4 g powdered milk + 100 ml PBS-Tween; PBS-Tween: 1 L PBS pH 7.4 + 1 ml Tween) for 30 min at 37 °C. Then, the blocking buffer was discarded, and 100 μl of serum was added, followed by incubation for 30 min at 37 °C. After washing the plates four times with PBS-Tween, 100 μl of a rabbit anti-sheep IgA antibody, conjugated to horseradish peroxidase (Serotec), at a dilution of 1/500 in PT-Milk, was added, followed by incubation for 30 min at 37 °C. The plates were then washed again four times with PBS-Tween and subsequently incubated in a peroxidase substrate and tetramethylbenzidine solution to produce a colour reaction, which was stopped by the addition of 50 μl of 2 M H_2_SO_4_. Finally, the absorbance was measured at 450 nm in a microplate reader (Titertek Multiskan). Positive and negative controls were included in every plate. Positive controls were obtained from a pool of serum from experimentally infected sheep with *T. circumcincta* and negative controls from non-infected sheep that were kept indoors. The results were expressed as the optical density ratio (ODR):

1 $$ \mathrm{O}\mathrm{D}\mathrm{R}=\left(\mathrm{sample}\ \mathrm{O}\mathrm{D}\hbox{-} \mathrm{negative}\ \mathrm{O}\mathrm{D}\right)/\left(\mathrm{positive}\ \mathrm{O}\mathrm{D}\hbox{-} \mathrm{negative}\ \mathrm{O}\mathrm{D}\right) $$

### Descriptive statistics

Descriptive statistical analysis for the two traits was conducted for the 529 sampled animals with the ‘pastecs’ package [22] in R [23]. The Shapiro-Wilk test was carried out to determine if the data for each trait was normally distributed. Due to the large number of zero counts in the FEC data and the fact that the animals graze during short periods of time (semi-extensive rearing system), we decided to use a ZINB model to estimate the zero-inflation parameter and then extended it to discriminate between exposed and unexposed animals. The zero inflated model with IgA data was compared to a simpler negative binomial model using a likelihood ratio test. Moreover, in this particular study, a zero inflated model is a biologically meaningful description of the system; the adverse climatic conditions for larval development of the year studied will reduce pasture contamination, and the short grazing periods due to the semi-extensive rearing system will reduce exposure, which means that some animals would not have been infected at the time of sampling, and may not have been infected since the last anthelmintic treatment. The zero inflated model also allows for a more natural extension into discriminating between infected and uninfected animals.

### Estimation of zero-inflation

In the zero inflated model, positive FEC are derived from a NB distribution, while a zero count can arise from either the NB distribution or the zero distribution (a binary distribution that generates structural zeros). The probability of belonging to the zero distribution is called the zero-inflation parameter. The animals that have zero counts arising from the zero distribution are assumed to have not been infected since the last anthelmintic treatment, so these animals can be excluded from further analysis. A Markov Chain Monte Carlo model similar to the one described in Denwood et al. [15] using the ‘*runjags*’ package [24] was employed to estimate the zero-inflation parameter.

**Fig. 2:**
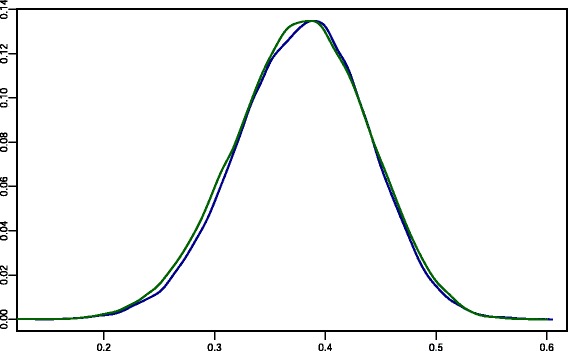
Posterior distribution obtained from the extended ZINB model. Each colour represents a different chain. Both chains have a mean around 0.38 and no sample was recovered from either of the chains with a zero-inflation parameter equal to zero (minimum value recovered = 0.12)

In this model, the negative binomial distribution arises from a gamma-Poisson mixture distribution. Uninformative priors were used for the parameters of the gamma distribution. The posterior distribution of the zero-inflation parameter is shown in Fig. 2.

### Extending the ZINB model

A zero-inflation model does not determine which animals are exposed and resistant (as opposed to unexposed). The classical ZINB model was therefore extended to accommodate IgA data as additional information for the animal status, i.e. infected or not recently infected. The animal status is calculated as,

2 $$ \mathrm{Status}=\Big\{\begin{array}{l}0;\kern1em \mathrm{not}\kern0.28em \mathrm{recently}\kern0.28em \mathrm{infected}\kern1em \mathrm{with}\kern0.28em \mathrm{probability}\kern0.28em 1-{P}^{\exp },\kern1em \\ {}1;\kern1em \mathrm{infected}\kern6em \mathrm{with}\kern0.28em \mathrm{probability}\kern0.28em {P}^{\exp}\end{array}\kern1em \operatorname{} $$

where status = 0 means that the animal has not been recently infected and status = 1 means that the animal is infected. *P* is the probability of being recently exposed and is equivalent to one minus the zero-inflation parameter. The raw egg counts (FEC/15) were used and it is assumed that for each animal *i*, the number of eggs counted arises from the following,

3 $$ \mathsf{Negg}{\mathsf{s}}_i\sim \Big\{\begin{array}{l}0\kern6em \mathrm{if}\kern0.28em \mathrm{Status}=0,\kern1em \\ {}\mathrm{Poisson}\left({\uplambda}_i\right)\kern1.48em \mathrm{if}\kern0.28em \mathrm{Status}=1\end{array}\kern1em \operatorname{} $$

where *λ*_*i*_ is the number of eggs arising from the gamma distribution (equation 4).

4 $$ {\uplambda}_i\sim \mathrm{gamma}\left(\mathrm{shape},\;\mathrm{rate}\right) $$

with the shape and the rate parameters of the gamma being calculated by the model. Similarly the IgA data can be partitioned in 2 gamma distributions (equation 5) based on the animal status.

5 $$ {\mathrm{IgA}}_i\sim \left\{\begin{array}{l}\mathrm{gamma}\left({\mathrm{sh}}_1,{\mathrm{rt}}_1\right)\kern2em \mathrm{if}\;\mathrm{Status}=0,\hfill \\ {}\mathrm{gamma}\left({\mathrm{sh}}_2,{\mathrm{rt}}_2\right)\kern2em \mathrm{if}\;\mathrm{Status}=1\hfill \end{array}\right. $$

with sh_1_, sh_2_, rt_1_ and rt_2_ being the two shapes and two rates respectively that parametrize the two gamma distributions. In the model, samples are drawn for sh_1_ and sh_2_ as well as for mn_1_ and mn_2_, which are the two means of the two gamma distributions. The rates are calculated by rate = shape/mean and the mean for the animals not recently infected (mn_1_) is always smaller than the mean of the infected (mn_2_). The fully annotated R code of the model is given in the Additional file 2.

The number of iterations sampled was 50,000, with the first 5,000 being discarded (burn in), and assessed convergence with the Gelman-Rubin statistic from the ‘*coda*’ package [25] being under 1.05.

Using the realisations of the animal status across the iterations (unexposed animals have status = 0, exposed and infected have status = 1), it is possible to calculate the probability for each animal to be in one status or the other, ${P}_i^{\exp }$; animals without zero FEC will always be in the infected status. The animals that were estimated to be unexposed, i.e. the animals with status = 0, in each sample of the Markov Chain were excluded from further analyses, allowing the use of simple statistical tools to analyse the remaining dataset for each sample.

### Correlations between phenotypes

Considering FEC, IgA and the realisations of animal status, ${P}_i^{\exp }$, the Kendall’s rank correlation coefficient was used to estimate the relationships among these three parameters. We used Kendall’s rank because it is an appropriate non-parametric hypothesis test. Correlations were calculated in R, using the ‘*ltm*’ package [26], for each sample of the Markov Chain and the average across the samples is reported below.

## Results

### Descriptive statistics of the phenotypic data

**Fig. 3:**
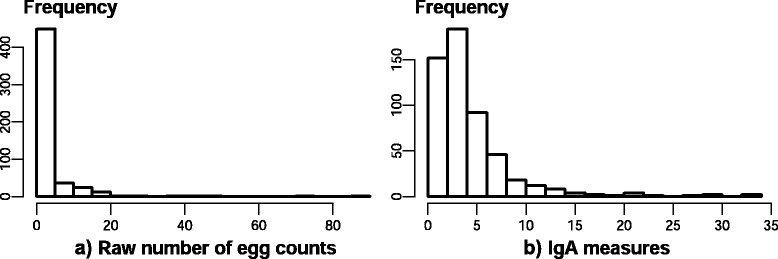
Distribution of (**a**) faecal egg counts and (**b**) plasma IgA across the 529 Spanish adult Churra ewes

*Faecal egg counts and larval identification*: Faecal egg counts of GIN ranged from 0 to 1,290 epg. In 64 % of the faecal samples no eggs were detected. The FEC mean and total variance were 38.2 (±105.9) and 11,218.9 respectively. The FEC distribution was heavily skewed to the right and showed a high level of over-dispersion (Fig. 3a). The Shapiro-Wilk test for the FEC data indicated a clear deviation from normality (*p*-value < 2.2 x10^-16^). Most of the eggs detected in positive samples were strongyle-type.

Apart from the GIN eggs, other parasite eggs were detected in faeces: 13.3 % of the sheep sampled had *D. dendriticum* eggs, with a range of 0–1,035 epg; 2.9 % had *Trichuris* spp. eggs (0–30 epg), two animals (0.9 %), had *Moniezia* spp. eggs (0–1,035 epg) and one ewe had *Capillaria* spp eggs at a concentration of 15 eggs per gram.

After collecting L3 from coprocultures, we identified the following genera of GIN: *Trichostrongylus* spp. (49.3 %), *T. circumcincta* (48.6 %), *Nematodirus* spp. (1.4 %) and *Cooperia* spp. (0.7 %). In all flocks, we confirmed the presence of *T. circumcincta*. We also observed a number of lungworm larvae, though they were not identified to the species level.

*IgA activity in the serum samples*: For individual animals, the mean ODR was 4.1 (±4.3), showing a range between 0.09 and 32.9; the ODR variance was 18.4. The distribution of IgA activities was positively skewed (Fig. 3b) with most of the sheep displaying relatively low IgA values, and only a few sheep presenting particularly high levels of IgA.

The Shapiro-Wilk test indicated a clear deviation from the normality (*p*-value < 2.2 x10^-16^). The Kolmogorov-Smirnov test indicated that the IgA was not gamma distributed (*p*-value = 0.0088), however this is due to the long tail of high IgA values. If the analysis is done with 10 animals less (effectively cutting the max IgA values to 20), the test indicates that the data is indeed gamma distributed (*p*-value = 0.21).

### Zero-inflation parameter and extension of the ZINB model for FEC data

**Fig. 4:**
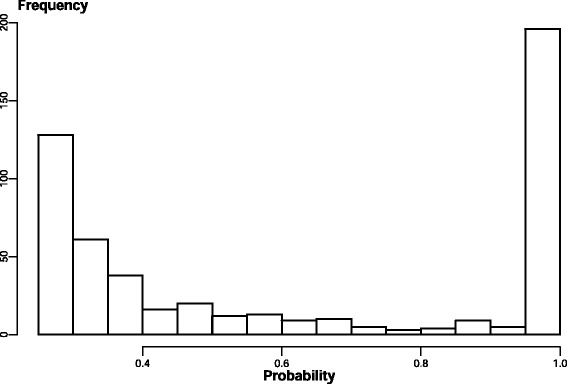
Probability of being exposed and infected, ${P}_i^{\exp }$, for the 529 animals sampled, which is calculated from the realisations of animal status (unexposed vs exposed) across the samples of the MCMC chain

To verify that the data is zero inflated, a likelihood ratio test was performed comparing the ZINB model to a simpler NB model, with a *p*-value of the likelihood ratio test = 6.62 x10^-5^, which indicates that the zero-inflated model provides a better fit to the data. The mean of the zero-inflation parameter was 0.38, this indicates that on average, 38 % of all the animals were not exposed and infected since the last anthelmintic treatment (2 months before the samples were taken), therefore it was estimated that 328 ewes were infected at sampling, even though only 190 had non-zero FEC. The zero-inflation parameter credible interval was much narrower when using the extended ZINB model as opposed to the ZINB model using FEC data only (from 0.013–0.46 to 0.25–0.49). The distribution of the probability of being exposed across all the animals in the data is shown in Fig. 4.

### Associations between phenotypes

The associations between phenotypes was calculated for the subset of animals that were considered exposed to the infection based on the implementation of the extended ZINB model (status = 1) in each sample of the Markov Chain. The correlations between Neggs, IgA and the estimated probability of being exposed to infection $\left({P}_i^{\exp}\right)$ are shown in Table 1. The phenotypic correlation between plasma IgA and number of eggs was close to zero and not statistically significant, while animal status was positively correlated to the number of eggs and IgA.

**Table 1:** Estimated correlations in the Churra sheep population

	Neggs	IgA	${P}_i^{\exp }$
Neggs	1	0.012	0.67**
IgA		1	0.18**
${P}_i^{\exp }$			1

Neggs is the number of eggs counted, IgA is the activity of IgA in serum (Optical density ratio) and ${P}_i^{\exp }$ is the probability of being exposed

**P < 0.001

## Discussion

Adult female sheep play a key role in the epidemiology of GIN infection because eggs deposited during the periparturient period influence the severity of the infection during the grazing season. However, outside the periparturient period, egg counts in adult sheep are typically low [27]. In general, GIN populations in naturally infected sheep are usually over-dispersed, with the majority of sheep showing low epg values and only a few sheep presenting a high level of infection [28]. In addition, some infected sheep will have low egg counts [8]. Therefore, supplementary information is needed as well as egg counts to determine which sheep are infected in adult sheep flocks.

In this study, the mean FEC per flock was quite low (38.2 epg) compared with other studies carried out in the same area (NW of Spain). Gutiérrez-Gil et al. [29] reported that the mean FEC was 260 epg between the years 1999 and 2003. Similar records were described by Martínez-Valladares et al. [30], who showed that the prevalence of GIN, based solely on the presence or absence of FEC, in sheep flocks was 100 %, and the mean epg was 237.2 (±375.9) between the years 2006 and 2011. In the current study, the low levels of infection are likely a consequence of the exceptional climatic conditions during this study since the longevity of infective trichostrongylid L3 nematodes is related to temperature and humidity [30, 31]. The table in Additional file 1 displays the mean temperature and precipitation for the period between December-June of the last 5 years (2007/2008–2011/2012) in the region of Castilla y León, highlighting the fact that the year 2011/2012 was extremely dry. According to Martínez-Valladares et al. [30], there is a direct relationship between GIN infection levels and the humidity of ambient air.

Faecal egg count, which has been for many years the traditional diagnostic tool for assessing GIN infection, has a low sensitivity [32], especially for very low counts as is the case in this study. Therefore, when the excretion of eggs in faeces is low, it is necessary to use other, more sensitive, diagnostic methods that might provide a more reliable indicator of infection.

IgA activity in the current study is moderately high, and this is presumed to be due to the fact that the antibodies persist for some time after GIN infection. The experimental studies of different breeds of sheep infected with GIN showed IgA activity for prolonged periods of time post infection. In an experiment carried out by Henderson and Stear [33], the peak of IgA was at 6–10 days after a deliberate infection with *T. circumcincta* in sheep although detectable IgA was evident 6 weeks later. Furthermore in an experiment with Churra sheep, Martínez-Valladares et al. [34] also showed that the elevated level of IgA in blood and nasal secretions was maintained 4 weeks post infection with this same parasite species. In the study by MacKinnon et al. [35] IgA activity was also evident 4 weeks post infection with *Haemonchus contortus* in Caribbean hair sheep.

In this study, a ZINB model was used to calculate the extent of zero inflation. This approach has been applied to several parasitic infections [15, 17, 18]. This model was then extended to identify the animals that are likely to be uninfected. This was done by adding the IgA information to the model. In a ZINB model using only FEC data, the model would not be able to assign animals with zero FEC as infected or uninfected. (Additional file 3).

There is heterogeneity among animals in the intensity of infection. Some infected animals will be exposed to more parasites than others. Both genetic variation in resistance and variation in exposure will contribute to the observed variation in IgA activity and FEC in exposed animals. Among animals that have not been exposed to parasites, FEC will be zero and parasite-specific IgA will be very low or zero. Animals with zero FEC and zero or low IgA activity are therefore more likely to be unexposed but it is possible that some of these animals have been exposed to low intensities of infection. Therefore the extension of the ZINB model to include additional data does not guarantee that every animal will be correctly assigned. It does however significantly improve the discrimination between exposed and unexposed animals and make subsequent analyses based on exposed animals more reliable (Fig. 4, Additional file 3).

To our knowledge, this is the first description of a ZINB model for the analysis of multiple traits with the aim of discerning which animals are infected and which have not been recently exposed or exposed to a very low infection level. This procedure is relatively straightforward and allows the study of nematode infections in adult animals and in flocks with low prevalence of infection, such as in Mediterranean dairy farms where animals are under a semi-extensive management system. The approach improves our ability to identify animals that have been infected with GIN, even at low FEC, which is needed for the study of host resistance in naturally infected individuals and the breeding of resistant sheep.

Because the over-dispersion pattern of GIN (number of eggs and adult worms found in the host) is also observed in other hosts such as cattle, free-range pigs, chickens, humans and wild animals [36–38], the approach described here could also be useful in other systems.

The correlations between the number of eggs and IgA and animal status were calculated using the non-parametric Kendall’s test. Although the number of eggs has been found negatively correlated with IgA in young lambs [39, 40], in the case of adult sheep, this correlation is not as clear and both Coltman et al. [39] and Gutiérrez-Gil et al. [29] reported non-significant correlations in naturally infected adult sheep after comparing logFEC and IgA against somatic antigen from *T. circumcinta* L4. Our results are similar, and suggest that this correlation is indeed close to zero in adult sheep. In experimentally infected adult sheep, Martinez-Valladares et al. [9] showed negative correlations between IgA in gastric mucus and FEC whereas the correlation between FEC and the serum IgA levels (which are lower than in the gastric mucus) were not significant. The absence of a clear correlation between plasma IgA and FEC may be a consequence of the fact that plasma IgA shows a complex relationship with mucosal IgA [41]. Alternatively, adult sheep may show greater IgE activity; reduced numbers of established parasites would decrease IgA responses and the relative importance of IgA on egg output would be lowered [42].

The extension of the ZINB model has allowed us to combine the information from two different traits that can indicate resistance or susceptibility to GIN. The IgA response was added to the model to help discriminate between unexposed and infected animals with zero FEC. Recent research has produced an index of the intensity of nematode infection in young lambs [43] and the observed correlations among the parasitological variable are necessary for this process. As mentioned previously, the use of a reliable indicator trait may be of interest not only for the management of parasite infections but also for the design of breeding programs aimed at achieving resistance to parasites.

## Conclusions

In summary, in the current study, two different phenotypes related to GIN infection (FEC and IgA against somatic antigen from L4 of *T. circumcincta*) were analysed. There was a high percentage of sheep without eggs in faeces (64 %) and a zero inflated model was used to detect the amount of zero inflation in the data. The ZINB model suggested that 38 % of sampled sheep had not been exposed to nematode infection in the previous 2 months, since the last anthelmintic treatment. Therefore, in addition to FEC data, the evaluation of IgA in serum may help to distinguish adult animals with low level of infection from resistant animals assist selective breeding for resistance to GIN.

## Abbreviations

ANCHE, National Association of Churra Breeders (Spain); FEC, faecal egg counts; GIN, gastrointestinal nematodes; IgA, immunoglobulin A; OD/ODR, optical density (ratio); ZINB/NB, (zero-inflated) negative binomial.

## Additional files

Additional file 1: Mean temperatures (ºC) and precipitation (mm) from December to June during the sampling period (highlighted in gray), and during the four previous years. (PDF 24 kb) Additional file 2: Annotated R code for the ZINB model. (PDF 20 kb) Additional file 3: Exposed probability in a classic ZINB model. Histogram of probabilities of being exposed for the data (a) and zoom of only the zero FEC (b) using only FEC data in the ZINB model. Animals with non-zero FEC will always have an “infected” status in the model (= 1) while animals with zero FEC can be exposed or unexposed. If only the FEC data is used, each animal with zero FEC will have a probability of being infected similar to one minus the zero-inflation parameter (b). (PDF 34 kb) Additional file 4: Raw data used. (TXT 5 kb)
