# Supplementary material for: Implementation of an extended ZINB model in the study of low levels of natural gastrointestinal nematode infections in adult sheep
Source: BMC Vet Res. 2016 Jun 10;12:97. doi: 10.1186/s12917-016-0723-7 (PMC4901511; doi:10.1186/s12917-016-0723-7)
Supplement: Additional file 1: — Mean temperatures (ºC) and precipitation (mm) from December to June during the sampling period (highlighted in gray), and during the four previous years. (PDF 24 kb) [file 12917_2016_723_MOESM1_ESM.pdf]

SUPPLEMENTARY MATERIAL 1

Supplementary Table S1. Mean temperatures (ºC) and precipitation (mm) from December to June during the sampling period (highlighted in gray), and during the four previous years.

Month	2007/2008		2008/2009		2009/2010		2010/2011		2011/2012	
	^{0}C	mm	^{0}C	mm	^{0}C	mm	^{0}C	mm	^{0}C	mm
December	1.9	11.4	2.9	50.3	3.7	110.4	3.1	92.5	3.8	14.0
January	4.5	33.2	2.7	38.1	3.4	62.1	3.9	42.4	2.4	12.2
February	6.3	32.5	4.3	21.2	3.6	59.3	4.5	28.5	1.9	5.8
March	6.5	16.6	7.5	12.5	6.2	55.3	7.3	41.1	8.0	9.8
April	9.5	68.4	8.6	28.4	11.0	37.6	12.2	43.2	7.7	72.7
May	12.2	100.3	14.9	22.6	12.3	36.0	15.3	36.7	14.8	35.8
June	16.9	34.1	18.7	30.5	16.9	68.5	17.6	22.6	18.8	14.8
