# Supplementary material for: Implementation of an extended ZINB model in the study of low levels of natural gastrointestinal nematode infections in adult sheep
Source: BMC Vet Res. 2016 Jun 10;12:97. doi: 10.1186/s12917-016-0723-7 (PMC4901511; doi:10.1186/s12917-016-0723-7)
Supplement: Additional file 2: — Annotated R code for the ZINB model. (PDF 20 kb) [file 12917_2016_723_MOESM2_ESM.pdf]

SUPPLEMENTARY MATERIAL 2

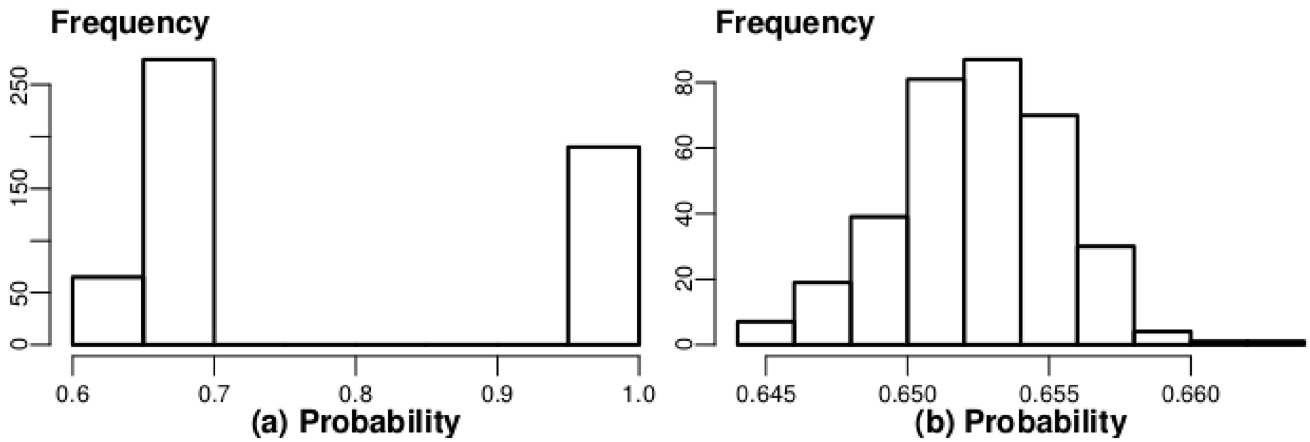

Figure SM2 – **Exposed probability in a classic ZINB model.** Histogram of probabilities of being exposed for the data (a) and zoom of only the zero FEC (b) using only FEC data in the ZINB model. Animals with non-zero FEC will always have an "infected" status in the model (= 1) while animals with zero FEC can be exposed or unexposed. If only the FEC data is used, each animal with zero FEC will have a probability of being infected similar to one minus the zero-inflation parameter (b).
