# Supplementary material for: Implementation of an extended ZINB model in the study of low levels of natural gastrointestinal nematode infections in adult sheep
Source: BMC Vet Res. 2016 Jun 10;12:97. doi: 10.1186/s12917-016-0723-7 (PMC4901511; doi:10.1186/s12917-016-0723-7)
Supplement: Additional file 3: — Exposed probability in a classic ZINB model. Histogram of probabilities of being exposed for the data (a) and zoom of only the zero FEC (b) using only FEC data in the ZINB model. Animals with non-zero FEC will always have an “infected” status in the model (= 1) while animals with zero FEC can be exposed or unexposed. If only the FEC data is used, each animal with zero FEC will have a probability of being infected similar to one minus the zero-inflation parameter (b). (PDF 34 kb) [file 12917_2016_723_MOESM3_ESM.pdf]

SUPPLEMENTARY MATERIAL 3

```
m <- “model {

for (i in 1 : nsheep) {
        Neggs[i] ~ dpois(lambda[i])
        lambda[i] <- lamb [i, status[i] + 1 ]
        status[i] ~ dbern(P) #animals status: 0 not recently infected, 1 infected
        lamb[i, 1] <- 0 #zero distribution for not recently infected
        lamb[i, 2]  dgamma(shape; rate) #gamma-poisson for infected

        IgA[i] ~ dgamma ( sh[i], rt[i] )  #IgA is gamma distributed
        #vector of means: position 2 exposed, 1 for non-exposed
        mn[i] <- vmn[ status[i] + 1]
        #vector of shapes: position 2 exposed, 1 for non-exposed
        sh[i] <- vsh[ status[i] + 1]
        rt[i] <- sh[i]/mn[i] #rate = shape / mean
}

# Prior zero-inflation
P ~ dbeta(1, 1)

#Priors Egg counts
shape ~ dgamma(0.001, 0.001)
p ~ dbeta(1, 1)
rate <- p/(1 – p)

# Priors IgA #
for(i in 1 : 2){
        unorderedmeans[i] ~ dgamma(0.001, 0.001) #uninformative means
}
vmn <- sort(unorderedmeans) #make sure uninfected mean is lower
vsh[1] ~ dgamma(0.001, 0.001)
vsh[2] ~ dgamma(0.001, 0.001)

#To avoid problems finding initial values
#inits# status, .RNG.seed, .RNG.name #initial values (animals status and RNG)
#data# FEC, IgA, nsheep #data used
#monitor# shape, rate, status, P, vmn, vsh # Outputs of the model
}”
```
